# Considerations in Recruiting Caregivers of Older Adults to Qualitative Internet-Mediated Research Using Facebook and Meta Business Suite

**DOI:** 10.1177/01939459251346583

**Published:** 2025-06-18

**Authors:** Steven Hall, Noelle Rohatinsky, Lorraine Holtslander, Shelley Peacock

**Affiliations:** 1 Faculty of Nursing, University of Alberta, Edmonton, AB, Canada; 2 College of Nursing, University of Saskatchewan, Saskatoon, SK, Canada

**Keywords:** social media, research participant recruitment, research ethics, caregivers, older adults

## Abstract

**Background:**

Internet-mediated research (IMR), increasingly prominent in social sciences and health care, uses online platforms for data gathering, offering cost-effectiveness and wide accessibility. Despite assumptions that older adults are less active on social media, recent trends indicate otherwise, with a notable presence on platforms like Facebook, making it a valuable recruitment tool.

**Methods:**

The Saskatchewan Caregiver Experience Study employed purposive maximum variation sampling to recruit caregivers via paid Facebook ads, manually shared Facebook posts, and community newsletters. Metrics such as reach, impressions, and link clicks from Facebook advertisements were used to evaluate recruitment effectiveness. Data quality was ensured through “one response per IP address” restrictions on SurveyMonkey.

**Results:**

We recruited 355 survey respondents who met the study inclusion criteria. Participants had a mean age of 60.9 years (range: 22-87). Paid Facebook ads were the most effective recruitment method, indicated by higher engagement and response rates. The 355 survey responses totaled 40 746 words, reflecting strong participant engagement. The absence of financial incentives in the study also likely improved data quality.

**Discussion:**

This method requires participants to have both device access and technological literacy. The study demonstrates the effectiveness of using social media for recruiting in qualitative research, highlighting its potential for inclusivity and representativeness, while also underscoring the importance of ethical considerations in IMR.

Internet-mediated research (IMR), a prominent methodology in contemporary social science and health care studies, leverages online platforms to gather data, and recruit participants for various research inquiries.^1^ IMR involves the use of online platforms and digital tools to conduct research, recruit participants, and collect data. This method offers numerous advantages, including its cost-effectiveness, wide reach, and accessibility, making it an appealing option for researchers across various disciplines.^2,3^ Recruiting participants for research studies can sometimes be a difficult task, with challenges such as limited access to target populations, time constraints, and budgetary restrictions.^4-6^ Recruitment challenges are especially true in qualitative research, where participants often need to fit focus groups or interviews into their schedules to be able to participate.^7^ IMR addresses many of these issues by tapping into the vast online communities and social networks in which individuals, including older adults, increasingly participate.^7^ One common misconception is that older adults are not active on social media platforms. However, recent data reveal a different reality. In Canada, for instance, roughly 1 in 3 individuals over the age of 65 is actively engaged in social media.^8^ Furthermore, platforms like Facebook, initially associated with younger users, have seen a shift towards a middle to older age demographic in recent years.^9^ Given the shifting demographics of Facebook users, this social media platform presents a feasible and economical method for recruiting research participants, including older adults and their caregivers.^10^

The Saskatchewan Caregiver Experience Study^11,12^ was a province-wide qualitative survey study that was hosted online on SurveyMonkey^13^ (SurveyMonkey Inc., San Mateo, CA, USA). The purpose of the Saskatchewan Caregiver Experience Study was to map the experiences and gather perspectives of caregivers on the challenges and positive aspects of caregiving in Saskatchewan^14,15^ and to identify their priority support needs.^16,17^ We recruited our participants through Facebook (by both manually shared posts and paid advertisements) and community newsletters that were distributed via e-mail. In this paper, we report the feasibility of our recruitment methods, as well as ethical considerations that were considered in both our execution of the study and in our application to the University of Saskatchewan Behavioural Research Ethics Board.

## Methods

The full detailed methodology for the Saskatchewan Caregiver Experience Study is published elsewhere.^12^ Purposive maximum variation sampling methods were used to recruit caregivers to older adults (aged >55 years for this study) via community newsletters, an online Facebook post shared manually in Saskatchewan community Facebook groups in which community members of Saskatchewan cities and towns share news updates and events, and a paid Facebook advertisement. Facebook posts were shared manually by the lead author (S.H.) joining several community groups and making posts in each individual group, while paid advertisements were promoted via machine learning algorithms, described herein.

Paid Facebook advertisements are a powerful method for businesses and individuals, and in this case, researchers, to reach specific audiences and drive various objectives.^18^ Advertisements can be targeted to audiences based on demographic information such as age, gender, and location.^18,19^ In the case of our study, we targeted our paid advertisement to individuals >40 years of age living in Saskatchewan, Canada. Once an advertisement is made live, Facebook’s machine learning algorithms continuously optimize its delivery to reach individuals who are most likely to take the desired action of the advertisement (ie, participate in a study). Machine learning is a system that learns as it receives new data without being explicitly programmed.^19^ Machine learning models predict a Facebook user’s likelihood of engaging with an advertisement based on the advertisement’s specific goal, such as increasing visits to a Web site or driving purchases.^19^ In the case of our study, the advertisement’s goal was to increase visits to and participation in our online qualitative survey. Over time, as more people view an advertisement, the Facebook algorithm improves its predictions on which users will interact with the advertisement, based on similar activities the users engage in both on and off Facebook.^19^ “On-Facebook” activities might include actions such as clicking on other advertisements or liking certain posts.^19^ “Off-Facebook” activities such as tracking which web sites users visit after using Facebook also facilitates the machine learning algorithm.^19^ Advertisers then receive detailed reports on the performance of their advertisements.^18^

Recruitment metrics were recorded based on where participants (both prospective and recruited) accessed the survey link from using unique “web link collectors” in the SurveyMonkey interface.^20^ Web link collectors allow SurveyMonkey users to track what channel their respondents are being recruited from, be it social media, e-mails, or newsletters.^20^ In our case, we had unique web link collectors for the community newsletters, manually shared Facebook posts, and paid Facebook advertisements. For the paid Facebook advertisements, more specific metrics were recorded using the Meta Business Suite, a “one-stop shop” where marketing and advertising activities on Facebook and Instagram can be managed.^21^ Meta Business Suite allows users to track insights and trends, see how posts are performing, and learn more about the audience seeing them.^21^ In Facebook’s Meta Business Suite, promotion can happen via a boosted post or an advertisement campaign. There are 3 terms specific to Facebook promotion: reach, impressions, and link clicks. *Reach* is how many unique Facebook users see a post in their newsfeed.^22^ *Impressions* are the total number of times the post is on screen in any newsfeed, including repeat views by Facebook users.^22^ *Link clicks* are how many Facebook users accessed the online qualitative survey.^22^

## Results

**Figure 1.**
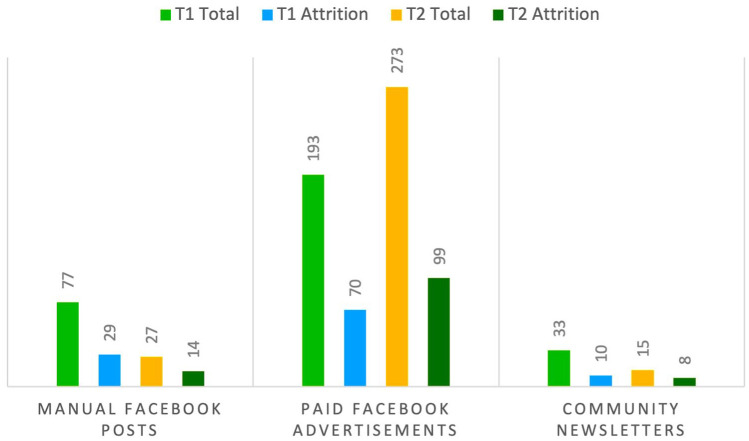
Comparison of outcomes from manually shared Facebook posts, paid Facebook advertisements, and community newsletters at T1 and T2.

**Figure 2.**
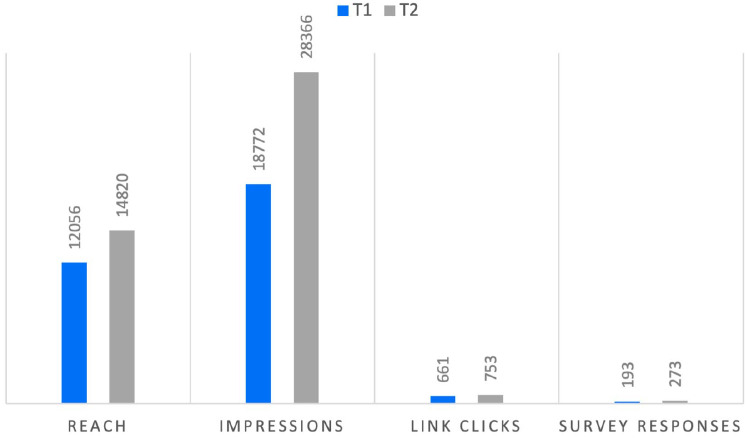
Metrics from paid Facebook advertisements at T1 and T2.

A total of 388 participants completed the survey, and 355 caregivers met inclusion criteria for the Saskatchewan Caregiver Experience Study.^12^ Participants had a mean age of 60.9 years, with a range from 22 to 87 years.^12^ Recruitment materials were promoted at 2 time points (T1 in June 2022 and T2 in July 2022). The recruitment metrics are presented in Figures 1 and 2. Paid Facebook advertisements were the most effective channel in terms of the survey responses and link clicks, indicating high engagement levels. T2 total (*n* = 273) had the highest survey response count, followed by T1 total (*n* = 193) for paid advertisements. The link clicks were also higher for T2 (*n* = 753) than for T1 (*n* = 651), suggesting either a broader reach or more effective targeting by Facebook’s algorithms at T2. Manually shared Facebook posts in community groups have no data on reach, impressions, or link clicks due to the fact Meta’s Business Suite was not used in these instances. Community newsletters had the lowest reported numbers among the 3 channels for survey responses. Overall, paid advertisements were the most effective recruitment channel, given their higher numbers for both link clicks and survey responses.

### Data Quality

Ensuring the authenticity of participants and the integrity of data is a significant challenge in IMR. Researchers must implement multistep screening processes to detect and prevent untrustworthy data, such as responses from bots.^23^ On SurveyMonkey, when an individual takes a survey, their IP address is recorded as metadata with the survey results.^24^ IP addresses can be traced to a single device, proxy server, or group of devices on the same network, but cannot be traced to an individual person,^24^ thus maintaining confidentiality. SurveyMonkey also has a setting where a survey can only receive 1 entry per IP address,^25^ which we chose to enable. In their overview of techniques to address threats to data quality, Jones et al^26^ noted that employing a “one response per IP address” technique will address the challenge of automated bot responses.

Another strategy that Jones et al^26^ highlights is the use of Completely Automated Public Turing Test to tell Computers and Humans Apart (CAPTCHA), a short task designed to be easy for humans but too complex for computers. However, a pitfall to CAPTCHA is that it is not available in all online platforms,^26^ which was also the case on SurveyMonkey at the time of our study. Furthermore, in our study there was no financial reimbursement, incentive, or reward for participants. Although recruitment in qualitative research can be enhanced with incentives, it can also increase the likelihood of fraud.^27,28^ We believe that the lack of incentive in our study likely contributed to the enhancement of data quality. It also certainly did not discourage participants from taking the time to share their caregiving experiences, as word counts from the 355 included responses totaled 40 746 words.^12^

## Ethical Considerations

### Public Trust in IMR

In IMR, there are concerns over privacy, confidentiality, consent, and public trust, due to unique challenges posed by the digital environment. The vast amount of personal information shared online, the ease of large-scale data collection, and the potential for unintended secondary uses all underscore the importance of responsible data handling.^29^ Additionally, the digital environment can blur the boundaries between public and private spaces, complicating individuals’ ability to anticipate or control how their data might be used.^29^ Furthermore, the use of digital traces of people’s online behaviors without consent has sparked debates about ethical practices in IMR.^30^ Concerns center on whether participants are adequately informed of the scope of data collection, the ways in which their data may be analyzed or shared, and the potential risks involved in large-scale data analytics.^31^ Researchers face the challenge of balancing methodological rigor with the obligation to protect participant autonomy, privacy, and well-being.^32^ As well, maintaining public trust in IMR requires responsible safeguards for privacy and ethical treatment of human data.^33^ Clear communication about data handling processes, transparent reporting of research aims and procedures, and robust security measures for data storage and transfer can foster a sense of confidence among participants.^29^ Adhering to evolving guidelines from ethics review boards can help ensure that IMR practices align with both ethical standards and public expectations.

### Case Example: Cambridge Analytica Scandal

The Cambridge Analytica scandal was a political and data privacy controversy that emerged in 2018, which majorly affected public trust. Cambridge Analytica, a British political consulting firm, had improperly accessed the personal data of millions of Facebook users without their consent via data harvesting.^34,35^ Up to 87 million Facebook users were affected, where their personal data were used for political advertising purposes without their consent.^34^ The scandal is an example of data misuse, which raised significant concerns over privacy and confidentiality in online data collection.^34-36^ As such, ensuring the security of data transmission and storage is critical, as is maintaining participant anonymity. The use of Internet data collection tools that have institutional licenses and have been preapproved by research ethics boards (REBs), such as SurveyMonkey^13^ in our study, can help in this case.

The Cambridge Analytica scandal also highlights the importance of maintaining high ethical standards to ensure participant safety in research practices. Obtaining and documenting informed consent in online research settings is more complex. The process needs to be tailored to ensure participants fully understand the study in which they are participating.^37^ Electronic consent methods require careful design to ensure accessibility and comprehension, as well as security.^5,37^ Being explicit about information on the benefits and risks associated with participation in the study is a key recommendation in collecting data via Internet tools, which also can enhance participant trust in the study at hand.^5^ Overall, privacy and confidentiality concerns in IMR stem from the challenges related to data protection and consent. Addressing these concerns requires close adherence to ethical guidelines and secure data practices.

### Research Ethics Boards

The expansion of REBs in behavioral sciences in the early 2000s led to what Haggerty^38^ termed as “ethics creep,” reflecting the intensification of external regulation of research activities.^39^ REBs play a critical role in reviewing protocols for IMR studies. All research involving human participants requires review and approval by REBs (in accordance with recognized international standards, including the Declaration of Helsinki and the Belmont Report), ensuring the protection of participants’ rights and welfare. However, the process of obtaining ethics approval for IMR studies can be time-consuming. Although these rigorous procedures are a necessary safeguard for any study with human participants, IMR research can face particular challenges related to digital privacy and consent, which may further extend the process. It often requires repetitive back-and-forth efforts for researchers and REBs, posing significant challenges to research timelines and budgets.^40^ As such, there is often hesitancy on the part of the researchers to conduct IMR studies, stemming from a range of issues including bureaucratic challenges related to the complexity and variability of the ethics review process, concerns over privacy and consent in digital research, and the aforementioned need to maintain public trust.^33^ Researchers must navigate these challenges while seeking to uphold the integrity of their work and the ethical standards of their field. Therefore, providing ample information to the REB with careful attention to how privacy, confidentiality, and informed consent will be ensured is a key strategy when applying to a REB for an IMR study. We detail our procedure of how we proposed our study to a behavioral REB herein.

#### REB application procedure

In the execution of this study, careful ethical considerations were critical to align with the guidelines provided by the University of Saskatchewan Behavioural REB. Regarding approaching the community Facebook groups, we outlined our planned process step-by-step to the REB. Our first step was iterating that we would initially contact the community Facebook group administrators and request permission to share our recruitment materials. We provided a drafted message that was sent to administrators through a dedicated caregiver research Facebook account, to ensure separation from the researcher’s personal inbox. The message described the purpose of the study, as well as the benefits and risks to participation (eg, health and social policy change as a benefit; psychological distress due to reflecting on a potentially challenging life experience as a risk).

The data collection in this project involved Internet-based interactions through the administration of an online survey via SurveyMonkey. The University of Saskatchewan’s institutional license for SurveyMonkey ensured encrypted connections and data security. To safeguard participant well-being and confidentiality, we outlined the consent form process. When the participant accessed the SurveyMonkey web collector link, they were directed to the consent form. They then had the opportunity to review the consent form, and at the bottom of the form, a button was presented, which said “Continue to Survey.” It was described that pressing this button indicated the participant was providing informed consent. Demographic collection forms within were designed to maintain anonymity, with participants only providing the first 3 characters of their postal code for data presentation purposes (Figure S1 presents a map showing the diverse geographic distribution of our participants due to this recruitment method). The potential risks for participants included psychological and emotional distress due to reflections on caregiving experiences. Mitigation strategies included providing supportive contact information, including toll-free telephone help lines for the Saskatchewan Health Authority and Alzheimer’s Society of Saskatchewan, in the consent form and at the end of the survey. The benefits of the research, centered on informing caregiver support services, were considered to outweigh the potential risks, justifying participant involvement.

Lastly, we acknowledged the ethical considerations of requesting participants’ time and personal information without offering financial compensation. We recognized that monetary incentives can encourage participation and acknowledge the value of participants’ contributions. However, the minimal-risk nature of this study and the broader benefits for informing caregiver support services were deemed sufficient to justify proceeding without direct compensation.

## Discussion

When manually posting in community Facebook groups, the outreach efforts for participant recruitment were carried out at no financial cost. However, to broaden the reach, the Saskatoon Council on Aging (a community partner in the study) allocated modest funds ($200 CAD) to enhance these efforts through paid advertising. The strategy of employing Facebook’s paid advertisement features is not without precedent. For instance, Ali et al^41^ previously demonstrated the effectiveness of such an approach. In their research, they invested $906 USD in Facebook advertisements to disseminate a web-based survey, reaching over 236 000 individuals and generating close to 9610 interactions with the survey link.^41^ Building on this supported method in the Saskatchewan Caregiver Experience Study, we spent a total of $200 CAD for 2 targeted advertisement campaigns on Facebook, specifically aiming to engage the Saskatchewan community. The investment paid off with the advertisements reaching 26 876 individuals. This reach translated into 1414 engagements. Notably in our study,^12^ a significant proportion of the respondents (58.3%) were persons aged 60 and above, underscoring the platform’s utility in engaging an older demographic.

The strategic use of social media advertising was a cost-effective method for recruiting participants, allowing for a wide distribution of the research initiative, and facilitating the involvement of a substantial number of potential participants (*N* = 355). The success of these campaigns is evident in the significant number of link clicks, suggesting that social media platforms, and Facebook in particular, can be powerful tools for reaching diverse audiences, including the older adult population which is often underrepresented in research. The ability of Facebook advertising to tailor content to specific demographics, locations, and interests makes it a valuable asset in the recruitment process, providing researchers with a direct line of communication to their desired study population. This approach enhances the visibility of the research and contributes to an inclusive and representative sample.

The ethical challenges and considerations highlighted in this study are reflective of a broader, evolving landscape within IMR. REBs have become increasingly vigilant in the face of the unique challenges posed by digital research methods. This heightened scrutiny is a response to the diverse and sometimes unpredictable nature of digital research environments.^39^ Researchers are required to navigate the delicate balance between innovation in research methods and adherence to ethical standards, a process often fraught with bureaucratic hurdles and potential delays.^40^ In the context of IMR especially, ethical considerations extend beyond traditional boundaries due to the public/private dichotomy of online spaces. The debate on what constitutes private versus public information online is ongoing, with significant implications for consent and privacy in digital research.^30^ Our study’s approach to obtaining consent and ensuring data security through encrypted platforms like SurveyMonkey is indicative of the efforts required to maintain participant anonymity and trust. Moreover, the ethical quandary of using digital traces without explicit consent raises fundamental questions about the nature of public data and the ethical responsibilities of researchers.^30^ In our study, we addressed these concerns by directly engaging with community Facebook group administrators for permission, thereby upholding ethical standards while also recognizing the communal nature of online spaces. The psychological and emotional risks associated with participation highlight the importance of ethical research practices, particularly in research involving personal experiences. Our mitigation strategies, such as providing supportive contact information, were essential efforts to ensure participant well-being. This approach aligns with the recommendations of Koene et al,^33^ emphasizing the necessity of safeguarding participant interests in IMR. Our study’s alignment with rigorous ethical standards, despite the challenges and complexities involved, not only reinforces the validity of our research but also contributes to the broader discourse on ethical practices in digital research.

### Strengths and Limitations

The online delivery of this study is a somewhat unconventional method of interaction in qualitative research, but its use has been emerging in recent years.^7,42,43^ Qualitative researchers have been increasingly turning to online platforms for data collection.^7^ Online data collection is particularly useful in research projects that seek to recruit participants in different geographical areas, as it eliminates the need for long-distance travel,^44^ which was an applicable strength in the Saskatchewan Caregiver Experience Study. Participants in this study did not have to travel to or host the researchers, and there were no incidental costs associated with participating in the research. Online data collection also is often more convenient for participants, as they can participate during their free time.^7^ Specifically for caregivers, online participation can be inviting because they do not need to arrange accommodations for the care recipient or respite time, which can often stand in the way of in-person research.^3,12,42^ Ethical challenges exist with online data collection, such as with ensuring informed consent is understood and the potential for participants to become distressed.^7^ These challenges were mitigated in this study by providing the consent form as the entry page to the survey and contact information for supportive services was provided at the beginning and end of the survey. A limitation in our data collection was that reach, impression, and link click metrics are not measured for the manually shared posts. This is a limitation in the features available on Facebook. To fully assess the effectiveness of our recruitment material dissemination, we would ideally need all data points for each channel, including reach and impressions for manual posts and community newsletters. This information would allow for a more complete assessment of each channel’s performance. Lastly, a limitation of the online delivery of the survey required participants to have access to a device to engage with the survey and also have the required technological literacy to complete an online survey.

## Conclusion

This study demonstrates the effectiveness and efficiency of IMR in recruiting caregivers to older adults, specifically utilizing Facebook as a primary recruitment tool. The high engagement rates in the Saskatchewan Caregiver Experience Study underscore the potential of social media platforms in reaching demographic groups across a wide geographic setting, as well as the often-underrepresented older adult population. The successful use of paid Facebook advertisements highlights the platform’s ability to target specific demographics, thereby ensuring a more inclusive and representative sample in research studies. In cases where funding for paid advertisements is not available, we would still recommend the method of manually sharing Facebook posts, as this still demonstrated effectiveness in our study. This approach to recruitment is not without its challenges. The ethical considerations inherent in IMR, particularly in relation to consent, privacy, and the use of digital data, are complex and require careful navigation. Moreover, the study showcases the advantages of online data collection in qualitative research, offering convenience for participants and reducing geographical and logistical barriers. While this approach enhances accessibility and inclusivity, it also highlights the need for technological access and literacy among participants, an important consideration for future research designs.

Building on these findings, a key methodological takeaway is the importance of balancing cost-effective recruitment tools that have the potential for a wide reach, in addition to rigorous ethical oversight. Researchers must navigate the complexities of digital consent, protect participant privacy, and ensure data quality through strategies such as IP address screening and the careful management of online advertising parameters. Furthermore, there is an ongoing need to address disparities in technological literacy and access, which can inadvertently exclude certain demographics in IMR. By carefully planning for these challenges along with transparent communication with REBs, thoughtful consent procedures, and robust data security measures, future qualitative research can harness social media platforms to recruit representative and diverse participants. The lessons learned in the undertaking of the Saskatchewan Caregiver Experience Study highlight the evolving nature of IMR. While digital tools streamline recruitment and expand reach, researchers must remain vigilant in upholding ethical standards, fostering participant trust, and adapting methodologies to an ever-changing online environment.

## Supplemental Material

sj-pdf-1-wjn-10.1177_01939459251346583 – Supplemental material for Considerations in Recruiting Caregivers of Older Adults to Qualitative Internet-Mediated Research Using Facebook and Meta Business Suite Supplemental material, sj-pdf-1-wjn-10.1177_01939459251346583 for Considerations in Recruiting Caregivers of Older Adults to Qualitative Internet-Mediated Research Using Facebook and Meta Business Suite by Steven Hall, Noelle Rohatinsky, Lorraine Holtslander and Shelley Peacock in Western Journal of Nursing Research
