# Supplementary material for: Considerations in Recruiting Caregivers of Older Adults to Qualitative Internet-Mediated Research Using Facebook and Meta Business Suite
Source: West J Nurs Res. 2025 Jun 18;47(9):879–86. doi: 10.1177/01939459251346583 (PMC12329150; doi:10.1177/01939459251346583)
Supplement: sj-pdf-1-wjn-10.1177_01939459251346583 – Supplemental material for Considerations in Recruiting Caregivers of Older Adults to Qualitative Internet-Mediated Research Using Facebook and Meta Business Suite [file sj-pdf-1-wjn-10.1177_01939459251346583.pdf]

Figure S1. *Geographic distribution of participants mapped using postal codes collected on the survey's demographic form. Figure originally printed in Canadian Journal of Nursing Research.*

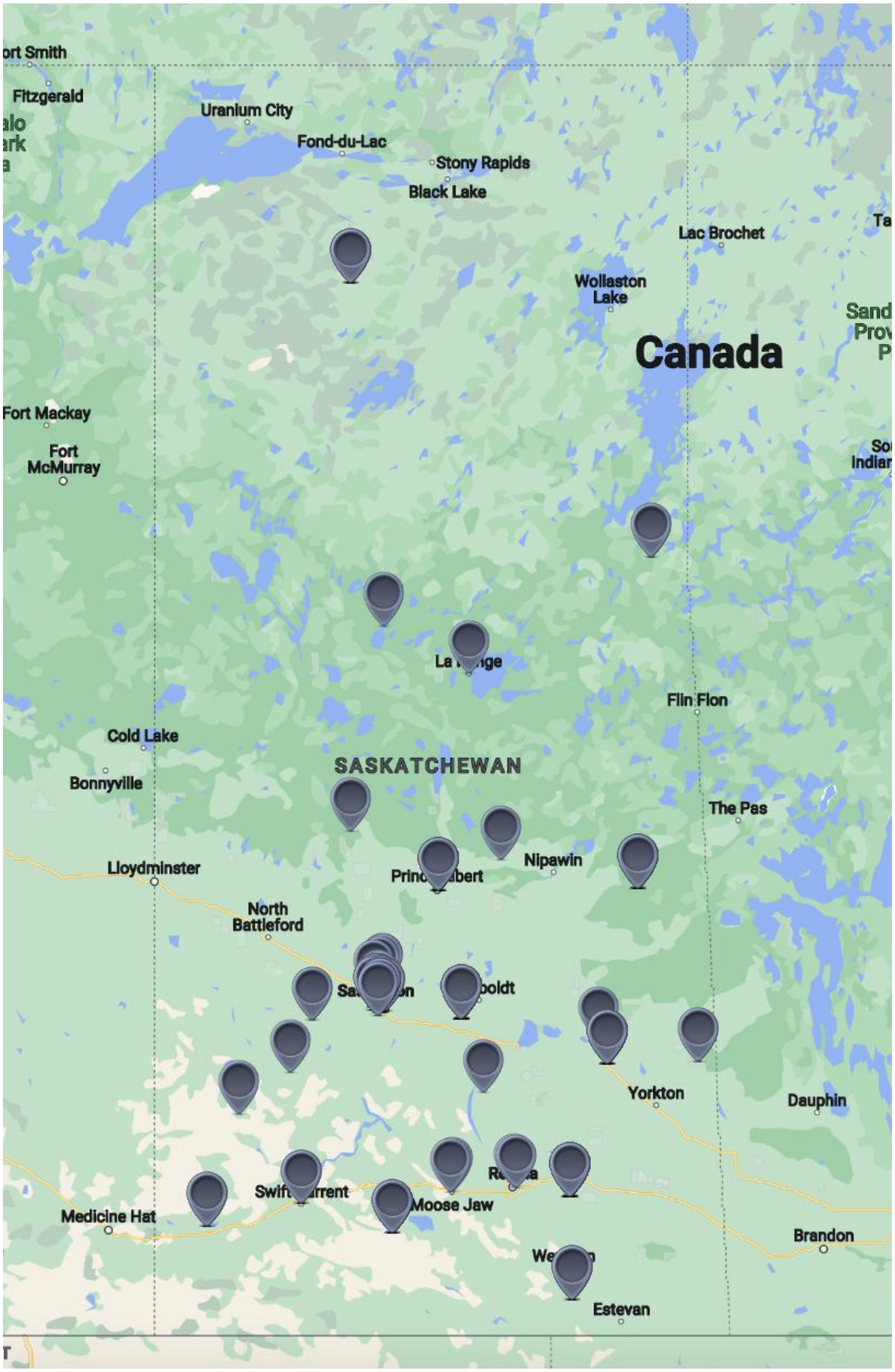

Source: Hall S, Rohatinsky N, Holtslander L, Peacock S. Mapping the Caregiver Experience in a Canadian Province: Research Methodology for the Saskatchewan Caregiver Experience Study. *Canadian Journal of Nursing Research*. 2024;56(3):234-246. doi:10.1177/08445621241227720
